# The Add-On Effect of Fluorouracil, Epirubicin, and Cyclophosphamide Regimens for Neoadjuvant Chemotherapy in Human Epidermal Receptor 2 (HER2)-Positive Breast Cancer: A Single-Center Retrospective Study

**DOI:** 10.7759/cureus.48255

**Published:** 2023-11-04

**Authors:** Ryusei Yoshino, Nana Yoshida, Nanami Ujiie, Masaki Nakatsubo, Mishie Tanino, Masahiro Kitada

**Affiliations:** 1 Thoracic Surgery and Breast Surgery, Asahikawa Medical University Hospital, Asahikawa, JPN; 2 Diagnostic Pathology, Asahikawa Medical University Hospital, Asahikawa, JPN

**Keywords:** pertuzumab, prognosis, neoadjuvant chemotherapy, her2, breast cancer

## Abstract

Background: The addition of pertuzumab to trastuzumab in neoadjuvant chemotherapy (NAC) for anti-human epidermal receptor 2 (HER2) positive breast cancer has shown a significant improvement in the pathologic complete response (pCR) rate. However, the add-on effect of an anthracycline-based regimen (standard-of-care regimen) remains unclear. In this retrospective, observational study, participants received pertuzumab combination therapy as NAC for HER2-positive primary breast cancer.

Methods: This study was conducted from January 1, 2020, to December 31, 2022. Patients who had not received at least three courses of pertuzumab owing to adverse events or those who had received preoperative radiotherapy were excluded.

Results: The pCR rate was 35.3% (12/34 patients). The pCR group had a significantly higher percentage of histopathologic grade III (1/11 patients, p=0.030) and a significantly higher percentage of hormone receptor-negative patients (7/12 patients, p=0.015) than the non-pCR group. The non-pCR group had a significantly higher incidence of vascular invasion than the pCR group (7/22 patients, p=0.036). Menopausal status, stage, and ki-67 values were not significantly different between the two groups.

Conclusions: This study suggests an unlikely add-on effect of an anthracycline-based regimen for NAC in HER2-positive breast cancer. Moreover, our results support that the pCR rate is high in patients with hormone receptor-negative, HER2-positive breast cancer.

## Introduction

Breast cancer accounts for high mortality among women and is a research field currently attracting global attention. In addition to surgery, patients with breast cancer are treated with hormonal therapy, anti-human epidermal receptor 2 (HER2) therapy, or chemotherapy, depending on the subtype. These are to be combined according to the circumstances. The goal of treatment is to achieve a cure and long-term survival through local and systemic therapy to the primary tumor and axillary lymph nodes where the cancer is considered to have progressed based on preoperative diagnosis. For prognosis, in addition to hormone receptors and HER2, multigene assays such as Oncotype DX, BRCA1/2 gene, and PD-L1 expression are used as markers. Clinical stage, grade, hormone receptors, HER2, and Ki-67 are commonly used, although some prognostic predictions are difficult and unclear. Surgical treatment options include mastectomy or breast-conserving surgery, often combined with radiation therapy, depending on the situation. In addition, sentinel node biopsy and axillary lymph node dissection are performed. Recently, breast reconstruction has been actively introduced.

HER2 is an oncogene with a structure similar to that of the epidermal growth factor receptor (EGFR) gene, which encodes the HER2 protein, a membrane-localized receptor with tyrosine kinase activity that is involved in epithelial cell growth and differentiation. HER2 amplification and overexpression occur in 15%-30% of all breast cancer cases, which are defined as HER2-positive breast cancer. By subtype, HER2-positive breast cancer has a poor prognosis, proliferates rapidly, and is prone to metastasis. However, anti-HER2 therapy for HER2-positive breast cancer is reportedly very effective [1-3].

There have been various studies on neoadjuvant chemotherapy (NAC) for HER2-positive breast cancer. Preoperative chemotherapy now offers the opportunity to increase the likelihood of breast conservation and allows better prediction of the prognosis in patients who were scheduled for mastectomy. The addition of pertuzumab to trastuzumab has significantly improved pathologic complete response (pCR) rates [4,5]. However, the add-on effect of an anthracycline-based regimen (the standard-of-care regimen) on the pCR, when combined with taxanes + trastuzumab + pertuzumab, has not been examined. Therefore, this study aimed to examine the effect of an anthracycline-based regimen combined with taxanes for NAC on the pCR rate.

Much of the research to date has been in vain, and the molecular mechanisms responsible for resistance to anti-HER2 therapy in breast cancer remain unclear. Furthermore, biomarkers to accurately predict treatment response and risk of recurrence in patients after NAC or anti-HER2 therapy are lacking. We will report on this as well as on recent genetic studies with some literature review.

## Materials and methods

Study design

This study was a single-center, retrospective observational study.

Setting

This study included patients who received an anthracycline-based regimen combined with pertuzumab for NAC to treat HER2-positive primary breast cancer from January 1, 2020, to December 31, 2022, at the Department of Thoracic Surgery and Breast Surgery, Asahikawa Medical University.

Eligibility criteria

The inclusion criterion was a histopathological diagnosis of HER2-positive breast cancer after preoperative needle biopsy; these patients were also treated with NAC in combination with an anthracycline regimen and pertuzumab. An immunohistochemical (IHC) score of 3+ or an IHC score of 2+ plus positive fluorescence in situ hybridization (FISH) results were considered to indicate HER2-positive breast cancer. Patients who did not receive at least three courses of pertuzumab for NAC because of adverse events (AEs) were excluded from the study. Furthermore, patients who had received preoperative radiotherapy (PASSION trial) were excluded.

Exposure and control groups

Patients with HER2-positive breast cancer received NAC consisting of four courses of epirubicin 90 mg/m^2^ + cyclophosphamide 600 mg/m^2^ + 5-fluorouracil 600 mg/m^2^ (FEC) every three weeks, followed by four courses of pertuzumab 420 mg/m^2^ (loading dose, 840 mg/m^2^) + trastuzumab 6 mg/kg (loading dose, 8 mg/kg) + docetaxel 75 mg/m^2^ every three weeks.

Ethical considerations

The requirement for informed consent was waived to ensure data anonymity. The Asahikawa Medical University Research Ethics Committee approved this study on January 18, 2023 (approval no. 22115). The study utilized medical information that was available to the researchers, and precautions were taken to protect the privacy of the individuals involved by de-identifying the data.

Definition of variables

Estrogen/progesterone receptor positivity was defined as a J-Score of ≥2 (positive cells representing at least 1% of the total cells) or an Allred score of ≥3. HER2 positivity was defined as an IHC score of 3+ or an IHC score of 2+ plus positive FISH results. For the histological response assessment, pCR was defined as ypT0/isN0. For one patient with bilateral breast cancer, the result of the side with the worst histological response was adopted.

Statistical methods (descriptive statistics and tests)

Median, minimum, and maximum values were calculated for continuous variables such as age and BMI. Continuous variables were analyzed using the D'Agostino-Pearson normality test. Comparison of parameters (menopausal status, stage, grade, hormone receptor status, HER2, Ki-67, vascular invasion, and lymph node metastasis status) between the pCR and non-pCR groups was performed using Fisher’s exact test using GraphPad Prism 9 software (GraphPad Software, San Diego, CA). Post hoc power analysis was used to analyze the post hoc power. We calculated event-free survival (EFS) after NAC for HER2-positive breast cancer using Kaplan-Meier curves in survival analysis. The starting point was set as the date of surgery performed after the completion of NAC, and the endpoint was set as the date of observation termination. Data of participants who were alive at the end of the study were censored. The definition of events included recurrence, metastasis, and death that occurred after surgery following the completion of NAC. No other statistical methods were used in this study. Moreover, two-sided p values were used for group comparisons; p<0.05 was considered a significant difference.

Missing values

In preoperative needle biopsies, the grade in three patients and Ki-67 values in two patients were difficult to determine or could not be measured. In addition, AE assessment was not performed in one patient because NAC was performed at another hospital.

## Results

**Figure 1:**
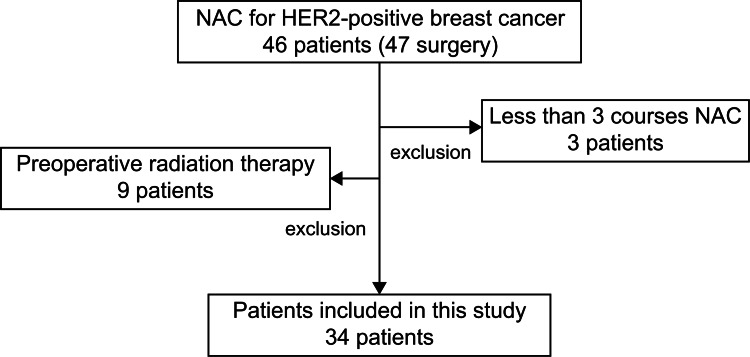
Patient selection NAC: neoadjuvant chemotherapy, HER2: human epidermal receptor 2

In total, 34 patients were enrolled in this study. Although 46 patients had received NAC for HER2-positive breast cancer, nine patients were participating in the PASSION trial, and three patients had received fewer than three courses of pertuzumab-based combination therapy; thus, these 12 patients were excluded from the study (Figure 1).

Table 1 shows the characteristics of the study participants. All patients were female, and the median patient age was 56 years (range, 30-74 years). The histopathological type of the tumor was invasive ductal carcinoma in 31 (91.2%) of the 34 patients; of these 31 patients, four (13%) had grade I, 24 (77%) had grade II, and three (10%) had grade III cancer. Hormone positivity and negativity were observed in 24 (71%) and 10 (29%) of the 34 patients. HER2 positivity (3+) was observed in 32 (94%) patients, and HER2 positivity (2+) in two (6%) of the 34 patients. Breast-conserving surgery (BCS) (Bp+ SN or Ax) was performed in 23 patients (68%).

**Table 1:** Characteristics of study participants Ax: axillary lymph node dissection; BMI: body mass index; Bp: partial mastectomy; DCIS: ductal carcinoma in situ; DTX: docetaxel; FEC: fluorouracil, epirubicin hydrochloride, and cyclophosphamide; FISH: fluorescence in situ hybridization; HER2: anti-human epidermal receptor 2; LCIS: lobular carcinoma in situ; Ly: lymph duct invasion; NAC: neoadjuvant chemotherapy; Per: pertuzumab; SN: sentinel lymphadenectomy; Tr: trastuzumab; v: vascular invasion

Total patients	34
Age	
Median (years)	56
Range (years)	~30-74
Sex	
Male	0
Female	34
BMI	
Median	21.4
Range	~17.3-40.4
Menopause	
Pre	10
Post	24
Histopathological Type	
DCIS or LCIS	2
Microinvasive carcinoma	0
Invasive ductal carcinoma	31
Special	1
Others	0
Right and left	
Right	14
Left	20
Grade (n=31)	
Ⅰ	4
Ⅱ	24
Ⅲ	3
Hormone receptor	
Positive	24
Negative	10
HER2	
3+	32
2+ (FISH positive)	2
Ki-67 (n=32)	
≥50%	9
<50%	23
Clinical tumor size	
T1	17
T2	13
T3	3
T4	1
Clinical nodal status	
N0	21
N1	11
N2	1
N3	1
Clinical stage	
ⅠA	11
ⅡA	14
ⅡB	4
ⅢA	3
ⅢB	1
ⅢC	0
Ⅳ	1
NAC (FEC→DTX+Per+Tr)	
Accomplish	33
3 courses	1
Surgical technique	
Bp+SN	21
Bp+SN (add Ax)	1
Bp+Ax	1
Bt+SN	8
Bt+SN (add Ax)	2
Bt+Ax	1
Ly	
Positive	5
Negative	29
V	
Positive	3
Negative	31
Postoperative histological assessment	
ypT0N0	10
ypTisN0	2
Others	22
Ejection fraction	
Average	64
Median	63
Range	~47-69

Table 2 reveals the results of the comparison between the pCR and non-pCR groups. The pCR rate was 35.3% (12/34 patients). The pCR group had a significantly higher proportion of grade III tumors (p=0.030) and hormone receptor-negative cases (p=0.015) than the non-pCR group. In contrast, the non-pCR group had a significantly higher incidence of vascular invasion than the pCR group (p=0.036). Thus, there were no significant differences in the menopausal status, stage, or ki-67 values between the two groups. The post hoc power was 0.99.

**Table 2:** Comparison between the pCR and non-pCR groups pCR: pathologic complete response, HER2: human epidermal receptor 2

	pCR (n=12)	Non-pCR (n=22)	p-value
Menopause			0.061
pre	1	9	
Post	11	13	
Stage			0.32
Ⅰ, Ⅱ	9	20	
Ⅲ, Ⅳ	3	2	
Grade			0.030
Ⅲ	3	0	
Ⅱ	8	22	
Hormone receptor			0.015
Positive	5	19	
Negative	7	3	
HER2			0.53
3+	12	20	
2+	0	2	
Ki-67			0.24
≥50%	5	4	
<50%	7	16	
Vascular invasion			0.036
Positive	0	7	
Negative	12	15	
Lymph node metastasis			0.14
Positive	0	5	
Negative	12	17	

The characteristics of AEs are presented in Table 3. Three patients discontinued NAC because of grade ≥3 AEs. Consequently, a decreased cardiac function, vomiting, and hemorrhagic gastric ulcer occurred in one patient each. Among the grade 1 or 2 AEs, anemia was the most common (78% [28/36 AEs]), followed by increased alanine transaminase in 18 (50%) and constipation in 15 (42%). Among grade ≥3 AEs, neutropenia was the most common (31% [11/36 AEs]), followed by anemia, febrile neutropenia, and decreased cardiac function (6% [2/36] each). Granulocyte colony-stimulating factor was used in 33% (12/36) of the AEs.

**Table 3:** Characteristics of adverse events (AEs) G-CSF: Granulocyte colony-stimulating factor

Adverse events (n=36)	Grades 1, 2	(%)	Grades 3, 4	(%)
Investigations				
Anemia	28	78%	2	6%
Neutrophil count decreased	7	19%	11	31%
Febrile neutropenia	0	0%	2	6%
Platelet count decreased	5	14%	0	0%
Alanine aminotransferase increased	11	31%	0	0%
Aspartate aminotransferase increased	18	50%	0	0%
Creatinine increased	4	11%	0	0%
Ejection fraction decreased	0	0%	2	6%
Gastrointestinal disorders				
Constipation	15	42%	0	0%
Diarrhea	4	11%	0	0%
Nausea	20	56%	1	3%
Vomiting	5	14%	1	3%
Mucositis oral	8	22%	0	0%
Gastric ulcer	0	0%	1	3%
General disorders and administration site Conditions				
Pain	6	17%	0	0%
Edema limbs	5	14%	0	0%
Infections and infestations				
Shingles	1	3%	0	0%
Skin and subcutaneous tissue disorders				
Dry skin	12	33%	0	0%
G-CSF used	12	33%		

**Figure 2:**
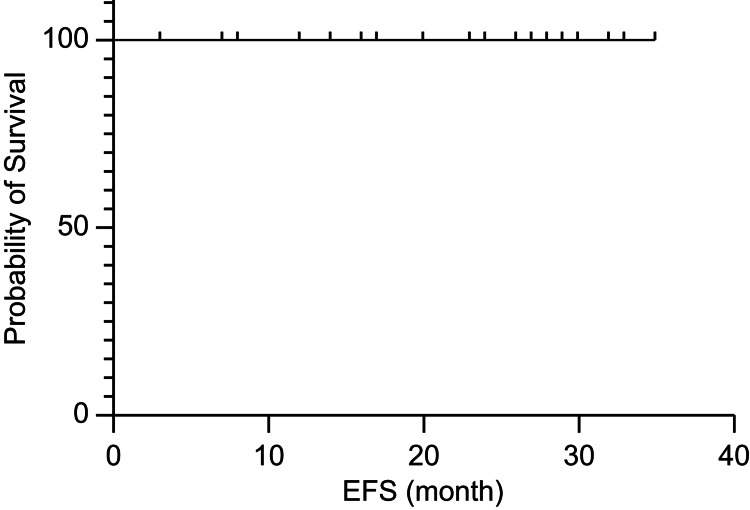
Event-free survival after neoadjuvant chemotherapy (NAC) for HER2-positive breast cancer NAC: neoadjuvant chemotherapy, HER2: human epidermal receptor 2, EFS: event-free survival

We calculated the EFS after NAC for HER2-positive breast cancer using Kaplan-Meier curves in survival analysis. Although the median follow-up period was 22 months (0-35 months), the EFS was 100%. During the follow-up period, we noted no recurrences, metastases, or deaths in any of the patients, and all patients were alive (Figure 2).

## Discussion

The pCR rate was 35.3% (12/34) in the group that underwent FEC as NAC for HER2-positive breast cancer. The pCR group had a significantly higher rate of grade III cancer than the non-pCR group (p=0.030). Moreover, the proportion of hormone receptor-negative patients was significantly higher in the pCR than in the non-pCR group (p=0.015). The non-pCR group had a significantly higher rate of vascular invasion than the pCR group (p=0.036). Menopausal status, stage, and ki-67 values were not significantly different between the two groups.

A recent study reported that paclitaxel and trastuzumab administered after fluorouracil, epirubicin, and cyclophosphamide (FEC) and NAC for the treatment of HER2-positive early-stage breast cancer resulted in a high pCR rate. In addition, several reports have shown that the combination of trastuzumab and pertuzumab improves pCR. However, there have been no definitive studies on the effect of anthracycline as an adjuvant to preoperative chemotherapy [6-8].

This study demonstrated the following two points. First, an add-on effect of anthracycline-based regimens and NAC is not expected in treating HER2-positive breast cancer. Second, a high pCR rate was observed in patients with hormone receptor-negative, HER2-positive breast cancer.

There is unlikely to be the add-on effect of an anthracycline-based regimen for NAC in treating HER2-positive breast cancer. Previous studies have reported pCR rates of 45.8% and 39.3% in patients treated with trastuzumab + pertuzumab + docetaxel in NAC [4,5]. In the TRYPHAENA study [9], the FEC regimen was used for NAC, and the pCR rate was 57.3%, suggesting the add-on effect of an anthracycline-based regimen. Although NAC was administered with the FEC regimen in this study, the pCR rate was low at 35.3% (12/34 patients). However, a possible reason for the low pCR rate in this study may be that several patients (71%; 24/34) were hormone receptor-positive, as discussed below. Therefore, preoperative chemotherapy with anthracyclines may not be expected to have an additive effect in HER2-positive breast cancer.

A high pCR rate has been observed in patients with hormone receptor-negative, HER2-positive breast cancer. Several previous studies [9-11] have concluded that patients with hormone receptor-negative, HER2-positive breast cancer are more likely to benefit from treatment. However, in this study, the proportion of hormone receptor-negative cases was significantly higher in the pCR than in the non-pCR group (p=0.015). This finding supports the report that the pCR rate favors hormone receptor-negative, HER2-positive breast cancer.

In this study, the pCR group had a significantly higher proportion of grade III cases than the non-pCR group (p=0.030), which is consistent with the results of a previous study [12]. However, the incidence of vascular invasion was significantly higher in the non-pCR than in the pCR group (p=0.036). Despite tumor reduction, this may have contributed to poor outcomes in the non-pCR group.

Grade ≥3 AEs included anemia (6%, 2/36), neutropenia (31%, 11/36), febrile neutropenia (6%, 2/36), decreased cardiac function (6%, 2/36), vomiting (1%, 1/36), and hemorrhagic gastric ulcers (1%, 1/36). In addition, three patients discontinued NAC because of decreased cardiac function, vomiting, and hemorrhagic gastric ulcers. Treatment discontinuation due to hemorrhagic gastric ulcers should be noted.

Recent studies have also identified potential markers of treatment sensitivity or resistance to therapy for HER2-positive breast cancer. This could aid in developing a standard of care for HER2-positive breast cancer and provide a range of combinations of anti-HER2 therapies. PI3K is a lipid kinase that mediates the phosphorylation of the inositol ring at position 3 of inositol phospholipids, a membrane component, which is controlled by the growth factor receptor. PI3K is a lipid kinase that mediates the phosphorylation of the inositol ring at position 3 of inositol phospholipids, a component of membranes. Among them, activation of the PI3K pathway by PIK3CA mutations may affect the prognosis of HER2-positive breast cancer. This suggests that PIK3CA mutations may predict resistance to the combination of NAC plus trastuzumab and lapatinib. The pCR rate after the combination of chemotherapy and anti-HER2 therapy was significantly lower in the mutant than in the wild-type PIK3CA group. This means that the presence of a PIK3CA mutation may identify a group that is less likely to benefit from anti-HER2 therapy [13,14]. Another area of interest is the presence of the FGFR gene, a protein found in cell membranes called the fibroblast growth factor receptor; FGFR gene abnormalities are already known to play a critical role in cancer cell growth, survival, and drug resistance. In addition, since FGFR gene aberrations are observed in various types of cancer such as lung cancer, gastric cancer, and brain tumors, it is expected to be a promising therapeutic target for cancer treatment. Moreover, recent studies have revealed that FGFR1 acquires resistance to lapatinib, trastuzumab, and TDM-1 in breast cancer. Furthermore, roblitinib, a selective inhibitor of FGFR4, was found to be potently effective against anti-HER2-resistant breast cancer [15]. These studies greatly support the design of future clinical investigations in the treatment of anti-HER2-resistant breast cancer.

In addition, the recent increase in the number of patients with noninvasive ductal carcinoma in situ (DCIS) has led to a strong interest in selecting high-risk DCIS patients who may require adjuvant therapy; the clinical and biological significance of HER2 overexpression in DCIS patients is not yet well defined. Therefore, according to the current NCCN guidelines for DCIS patients, HER2 testing is not recommended due to the unclear evidence of HER2 status as a prognostic factor in DCIS. However, accumulating evidence from recent studies suggests that the HER2-positive/hormone receptor-negative DCIS subtype has a poor prognosis, whereas patients with the HER2-negative/hormone receptor-positive subtype have the most favorable outcome [16]. This is another area where we expect further studies to accumulate.

This study has certain limitations. First, as this was a retrospective study at a single institution, the sample size was small, which may have caused random errors. Additionally, given that the follow-up period was short, the survival analysis results may be biased. Second, most previous studies included patients with a tumor size of T2 or larger; however, this study also included T1 cases. Finally, the results for patients not meeting the inclusion criteria are less likely to be generalizable. However, we believe this study is valuable because it evaluated the usefulness of NAC for HER2-positive breast cancer, which is currently under investigation.

## Conclusions

This study’s results suggest an unlikely add-on effect of an anthracycline-based regimen with NAC in treating HER2-positive breast cancer. This may have critical implications for selecting future NAC regimens considering FEC toxicity. Our results further support that the pCR rate is high in patients with hormone receptor-negative, HER2-positive breast cancer. Further accumulation of cases is crucial to fully explore the appropriate use of NAC in HER2-positive breast cancer.
